# Human Health Risks and Air Quality Changes Following Restrictions for the Control of the COVID-19 Pandemic in Thailand

**DOI:** 10.3390/toxics10090520

**Published:** 2022-08-31

**Authors:** Jenjira Kaewrat, Rungruang Janta, Surasak Sichum, Chuthamat Rattikansukha, Wittaya Tala, Thongchai Kanabkaew

**Affiliations:** 1 School of Languages and General Education, Walailak University, Nakhon Si Thammarat 80160, Thailand; 2 Center of Excellence in Sustainable Disaster Management, Walailak University, Nakhon Si Thammarat 80160, Thailand; 3 Environmental Science Research Center (ESRC), Faculty of Science, Chiang Mai University, Chiang Mai 50200, Thailand; 4 Environmental Chemistry Research Laboratory (ECRL), Department of Chemistry, Faculty of Science, Chiang Mai University, Chiang Mai 50200, Thailand; 5 Faculty of Public Health, Thammasat University, Pathum Thani 10120, Thailand

**Keywords:** coronavirus, air quality index, criteria pollutants, health risk assessment

## Abstract

The coronavirus (COVID-19) pandemic first impacted Thailand in early 2020. The government imposed lockdown measures from April to May 2020 to control the spread of infection. Daily lifestyles then morphed into a so-called new normal in which activities were conducted at home and people avoided congregation in order to prevent the spread of an infectious disease. This study evaluated the long-term air quality improvement which resulted from the restrictions enforced on normal human activities in Thailand. The air quality index (AQI) of six criteria pollutants and health risk assessments were evaluated in four areas, including metropolitan, suburban, industrial, and tourism areas in Thailand. The results showed that, after the restriction measures, the overall AQI improved by 30%. The subindex of each pollutant (sub-AQI) of most pollutants significantly improved (by 30%) in metropolitan areas after human activities changed due to the implementation of lockdown measures. With regard to industrial and tourism areas, only the sub-AQI of traffic-related pollutants decreased (34%) while the sub-AQIs of other pollutants before and after lockdown were similar. However, the changes in human activities were not clearly related to air quality improvement in the suburban area. The overall hazard index (HI) after lockdown decreased by 23% because of the reduction of traffic-related pollutants. However, the HI value remained above the recommended limits for the health of the adult residents in all areas. Therefore, strict regulations to control other pollutant sources, such as industry and open burning, will also be necessary for air quality improvement in Thailand.

## 1. Introduction

Coronavirus Disease 2019 (COVID-19) was found in Asia in late December 2019 and rapidly spread to every continent. It became a global health crisis from 2020 to the present. There have been 500 million cases and 6 million deaths across the world as of this study [1]. Effective medicines and vaccines were limited in the early stage of the pandemic. Many countries around the world enforced lockdown measures, seeking to control the spread of the virus by imposing many social restrictions on their citizens. This caused an unprecedented reduction in economic and logistic activity. Thailand’s first wave of COVID-19 in March 2020 began in Bangkok, and then spread to other provinces. Lockdown measures were imposed at the end of March 2020 when public transportation was restricted, business operations were suspended, and all international and domestic flights were canceled in addition to restricting interprovincial movements within Thailand. Additionally, the government ordered people to work at home and postpone their holidays to prevent the transmission of COVID-19 in workplaces and community spaces. After the implementation of these measures, the number of infections decreased to lower than 10 cases per day. Economic activities resumed on 1 May 2020 and continued until early April 2021, at which point there was a second COVID-19 wave in Thailand. The second time, the government employed a targeted strategy and imposed a lockdown on specific areas in addition to closing schools to prevent further spread of infections. Since the lockdown measures were implemented in order to control the spread of COVID-19, the daily life of Thai people changed, settling eventually into a new normal in which they adjusted their work, study, and business activities to working and studying at home via online platforms, online shopping and ordering food, and using online media for communication. These changes may have also served to support air quality improvements, in terms of source reduction.

Thailand faces air pollution, which results in adverse health effects, including increased risk of mortality and morbidity from respiratory infections, cardiovascular diseases, and lung cancer. Guo et al. [2] indicated that increasing air pollutant concentrations, including PM_10_ (10 mg/m^3^), O_3_ (19.6 µg/m^3^), and SO_2_ (2.62 µg/m^3^), were associated with increases in nonaccidental mortality in Thailand, by 0.40%, 0.78%, and 0.34%, respectively. Pinichka et al., [3] also showed that air pollutants contributed to 10% (NO_2_), 7.5% (PM_2.5_), and 3.4% (PM_10_), respectively, of all-cause mortality in Thai adults. Traffic congestion, industrialized areas, forest fires, and agricultural burning within and outside Thailand are major sources of air pollution in Thailand. The source of air pollution in each area varies spatially and temporally. Thailand normally uses the air quality standard for air quality control and air quality index (AQI) to warn the population about the health effects of exposure to air pollution. In many countries, including China, the USA, South Korea, Canada, Australia, and Mexico, the AQI is also used [4]. AQI descriptors and warning messages vary between countries, as do the groups that receive such warnings. The AQI is suitable for acute effect warnings, while the health risk assessment (HRA) is a proper methodology to estimate the chronic health effects of exposure to air pollution. The HRA can be estimated by the hazard quotient (HQ), which is usually used to evaluate the risk of noncarcinogenic pollutants. In addition, the HRA can also be used to assess the overall risk of disease from whole lifetime exposure and to forecast the expected health effect of policies on air quality management, which are critical to guiding public policy decisions [5]. In Thailand, use of the HRA to estimate the health risk of exposure to air pollutants remains limited, compared to other countries such as China, Bangladesh, and Malaysia, particularly after lockdown measures and daily life changed into the aforementioned new normal [6,7,8].

Many studies reported that air quality significantly improved due to the restrictions on human activities that were imposed during lockdowns [9,10,11]). Kaewrat and Janta [12] and Wetchayont [13] investigated air quality in Thailand after the implementation of lockdown measures, with both studies finding that air pollutants, particularly traffic-related pollutants, decreased due to the reduction of emission sources. Meanwhile, areas in which pollutants were associated with several emission sources saw no significant air quality improvement. These studies were undertaken in a short period after the lockdown measures. Moreover, the quality of ambient air was influenced both by emission sources and by meteorological factors. These affected the dilution, accumulation, and chemical reaction processes of pollutants emitted from local sources, and further affected the distribution and concentration of pollutants [14,15,16]. The present study, therefore, explored long-term air quality in metropolitan, industrial, tourism, and suburban cities of Thailand where the lifestyle changed to the new normal. Moreover, the human health risks from exposure to air pollutants during this new normal period were calculated. The results from this study served to provide information on air quality which should be useful for air quality management in a new normal, post-COVID era.

## 2. Materials and Methods

### 2.1. Description of Study Areas

**Figure 1:**
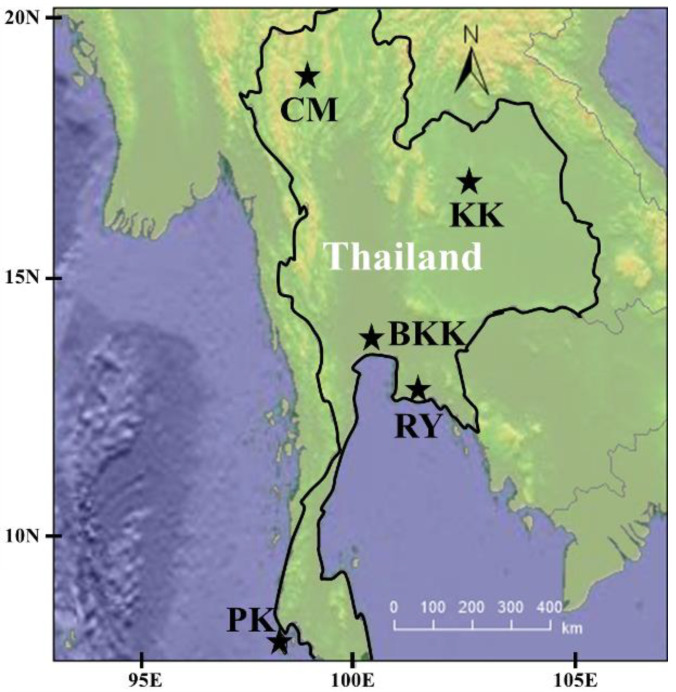
Study areas: Chiang Mai (CM); Bangkok (BKK); Khon Kaen (KK); Rayong (RY) and Phuket (KK).

The study was conducted in Thailand (Figure 1). Five provinces were selected based on the economy and major income of the population.

Metropolitan areas

Bangkok (BKK) (13°45′ N, 100°29′ E) is the capital city and economic center of Thailand. BKK is a metropolitan area with a high population density (about 4000 per km^2^) and an inadequate road and public transport network, which results in heavy traffic congestion in the city. Air quality data from Bangkok was obtained from air quality monitoring (AQM) stations installed in community areas near main roads. Transportation and open burning are major sources of air pollution in this area.

Chiang Mai (CM) is the largest city in northern Thailand (18°47′ N, 98°59′ E) and has a population density of about 300 per km^2^ (3000 per km^2^ in the city). Agriculture and tourism are major incomes in CM. Before COVID-19, Chiang Mai received over 10 million visitors per year. Traffic congestion has become a serious problem in the city of Chiang Mai due to rapid urbanization and an inadequate road system. The AQM station installed in the Chiang Mai City Hall is surrounded by communities, forests, and agricultural areas. Open burning and transportation are major sources of air pollutants in this area.

Suburban Area

Khon Kaen (KK) is one of four major cities in northeastern Thailand (16°26′ N, 102°50′ E.). The population density is 165 per km^2^ (2500 per km^2^ in the city). Agriculture, particularly sugarcane cultivation, is the major source of income in Khon Kaen province. Open burning is a major source of air pollution in this area. The AQM stations installed in the city are surrounded by communities and transportation areas. The main source of pollutants in Khon Kaen are community areas, open burning and traffic.

Industrial area

Rayong (RY) is located on the eastern coast of the Gulf of Thailand (12°40′ N, 101°16′ E). Rayong has a population density of 200 per km^2^. This province is known as the industrial heart of Thailand. Many industries are present in the province, including chemical, petrochemical, and automotive industries. Additionally, this province also has famous beaches that attract tourists. The industrial and tourism sectors are important for local incomes in the province and city. An AQM station is installed at the Rayong Provincial Agriculture Office, which is surrounded by community areas and is located 15 km to the west of the Map Ta Phut Industrial Estate. Both community and industrial emissions impact air quality in this area.

Tourism area

Phuket (PK) is the largest island in Thailand and is located on Thailand’s western coast in the Andaman Sea (7°53′17″ N, 98°23′51″ E). Phuket province has a population density of 755 per km^2^ while the city has a population density of 6600 per km^2^. Before COVID-19, Phuket normally received about 10 million tourists per year and tourism is an important sector of the Phuket economy. The AQM station is installed in a community area near main roads, as transportation is a major source of air pollution in this area.

### 2.2. Air Pollutant Concentration

The hourly concentration of criteria pollutants, including particulate matter ((PM_2.5_ and PM_10_), carbon monoxide (CO), ozone (O_3_), nitrogen dioxide (NO_2_), and sulfur dioxide (SO_2_)) and meteorological parameters, including temperature, wind speed, humidity, and amount of rain, were obtained from the AQM stations monitoring by the Pollution Control Department (PCD), Ministry of Natural Resources and Environment, Thailand. The PCD usually uses various techniques for air pollutants monitoring including Beta Radiation Attenuation and Tapered Element Oscillating Microbalance: TEOM (PM_10_ and PM_2.5_), UV Fluorescence (SO_2_), Chemiluminescence (NO_2_) and UV absorption (O_3_). The study was conducted from January 2018 to November 2021. The concentration was divided into two periods based on the implementation of lockdown measures: the period before lockdown (1 January 2018 to 31 March 2020) and the period after lockdown, or new normal period (1 June 2020 to 30 November 2021).

### 2.3. Air Quality Index (AQI)

Thailand’s AQI was developed by the Pollution Control Department and was used to investigate air quality in this study. AQI values were calculated from subindexes of criteria pollutants (sub-AQI) and combined into a single numerical value to indicate the level of an acute effect. Sub-AQI values of each pollutant were calculated based on Thailand’s AQI (PCD, 2020) and the highest sub-AQI was shown as the AQI for that day, for which the values can be classified into five classes as shown in Table 1.

### 2.4. Health Risk Assessment

Health risk assessment was estimated for non-cancer risk from inhalation. The hazard quotient (HQ), which is the ratio of potential exposure to pollutants and its level without adverse health effects, was used to assess the health risks of adult residents (19–75 years) from exposure to the six criteria pollutants during pre-COVID and post-COVID periods. Lina et al. [17] referred to the study of Limy in 1996 indicating the level of health hazards based on the HQ value as follows: no hazard (HQ values < 0.1); low hazard (HQ values of 0.1–1.0); moderate hazard risk (HQ values of 1.1–10); high hazard risk (HQ values > 10). The HQ value was calculated from Equation (1) [18]:

(1) $$\mathrm{HQ}=\frac{\mathrm{ADD}}{\mathrm{RfD}}$$

which is the average daily dose. ADD (mg/kg.d) is the exposure to pollutants by respiratory inhalation (mg/kg.d), and the reference dose (RfD) refers to an estimated level of human daily intake without adverse health effects during a lifetime (mg/kg.d). The RfD of pollutants was estimated from its value for the reference concentration (RfC) by Equation (2). Thailand’s standard for ambient air pollutants was applied to obtain the RfC value: PM_10_ (50 µg/m^3^), PM_2.5_ (25 µg/m^3^) CO (10.26 mg/m^3^), NO_2_ (57 µg/m^3^), SO_2_ (100 µg/m^3^) and O_3_ (140 μg/m^3^). The Inhalation rate: IR (m^3^/d) and Bodyweight: BW (kg) of each group are presented in Table 2.

(2) $$\mathrm{RfD}=\frac{\mathrm{RfC}\;\times \mathrm{IR}}{\mathrm{BW}}$$

The ADD of pollutants was evaluated from Equation (3) [19,20]. Details of each parameter are presented in Table 2.

(3) $$\mathrm{ADD}=\frac{\mathrm{CA}\;\times \;\mathrm{IR}\;\times \;\mathrm{ET}\;\times \;\mathrm{EF}\;\times \;\mathrm{ED}}{\mathrm{BW}\;\times \;\mathrm{AT}}$$

where CA is the concentration of air pollutants (mg/m^3^) which is calculated from an average of pollutant concentrations in both the summer and rainy seasons, IR is the inhalation rate of adult residents (19–75 year) (m^3^/h), ET is the exposure time (h/d), and EF is the exposure frequency (d/y). ED is the exposure duration (years), BW is the body weight of adult residents (19–75 year) (kg), and AT is the average time (d).

**Table 2:** Exposure factors used for calculation in this study.

Exposure Factors	Symbol	BKK	CM	KK	RY	PK	Reference
Mean concentration (mg/m^3^)							
CO	CA_CO_	0.339	N/A	0.684	0.543	0.308	This study
NO_2_	CA_NO_2__	0.013	0.015	0.018	0.013	0.014	This study
PM_2.5_	CA_PM_2.5__	0.020	0.028	0.028	0.016	0.019	This study
PM_10_	CA_PM_10__	0.035	0.044	0.052	0.028	0.038	This study
O_3_	CA_O_3__	0.036	0.054	0.061	0.045	0.043	This study
SO_2_	CA_SO_2__	0.007	0.002	0.009	0.004	0.002	This study
Inhalation rate (m^3^/h)	IR	0.89					[21]
Exposure time (h/d)	ET	24					
Exposure frequency (d/y)	EF	365					
Exposure duration (y)	ED	30					[21,22]
Bodyweight (kg)	BW	71.8					[21,22]
Average time (d)	AT	10,950					

The hazard index (HI) was also used to estimate the total noncarcinogenic risk from exposure to many pollutants at the same time, as calculated by Equation (4) [20,23].

HI = HQ_1_ + HQ_2_ + … + HQ_n_ (4)

where 1–n: specified pollutants in the air.

### 2.5. Clustering Analysis for Health Risk Assessment

Backward trajectory cluster (path) analysis is normally used to determine the source regions of pollutants. The trajectory also presents the association of pollutant concentration in the air arriving in a receptor area. This study applied air mass trajectories to indicate the potential of sources and areas on adverse health effects resulting from long-term exposure to the air pollutants in each area. The pollutants are normally moved to the receptor area via the movement of the air, an average pollutant concentration of the clustered trajectories presents the concentration of the pollutant in each path. Additionally, a number of trajectories in each cluster indicated the exposure frequency in the health risk assessment.

The backward trajectory of each study area was calculated by the Hybrid Single-Particle Lagrangian Integrated Trajectory (HYSPLIT) model TrajStat Trajectory Statistics function. The TrajStat function, which was developed and published by Wang et al., [24] is a useful tool for clustering trajectories in source identification. The trajectory model’s meteorological input was the Global Data Assimilation System (GDAS) and meteorological data (1° × 1°). A 24 h backward trajectory was calculated twice per day (08:00 am and 08:00 pm Local Sidereal Time (LST)) at 10 m above ground level (AGL). A total of 1096 trajectories (June 2020–November 2021) per study area were clustered using the TrajStat application. The clustered trajectories (path of air movement) represented the direction of the pollutant’s source region, while the number of trajectories indicated the frequency with which residents were exposed to the pollutant. The exposure frequency (EF) of each cluster was calculated by Equation (5) [25]. Then, the HI value of each cluster was calculated by Equation (4).

EFi = %Trajectory cluster i × 365(5)

where EFi is the exposure frequency of cluster i and %Trajectory cluster i is the percentage of trajectories in cluster i.

## 3. Results and Discussion

### 3.1. Concentrations of Air Pollutants

**Figure 2:**
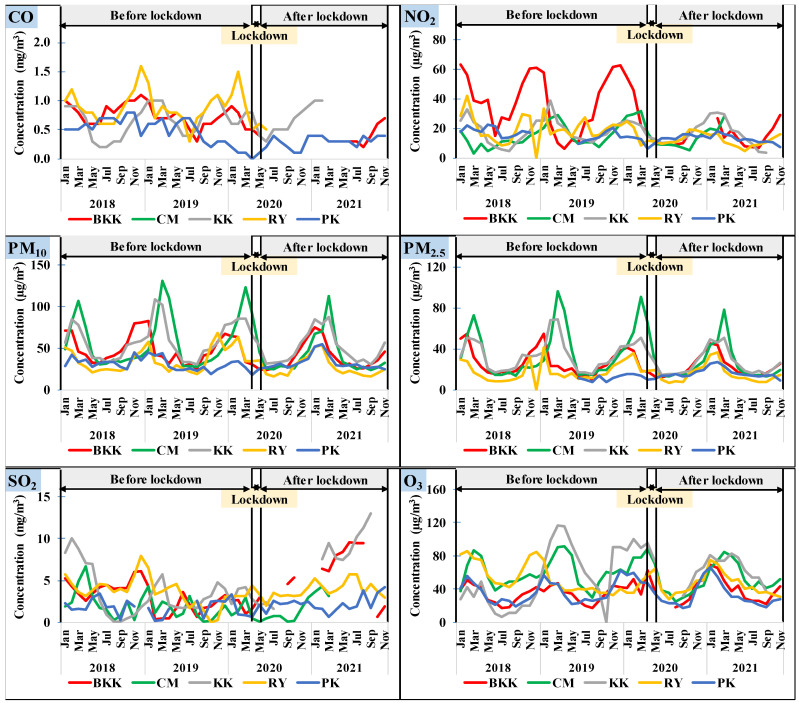
Pollutant concentration before lockdown, in lockdown, and after lockdown periods in Thailand.

Thailand’s government imposed rigorous social measures and a full-scale national lockdown from 1 April to 31 May 2020 in order to control the COVID-19 pandemic. After the lockdown measures were relaxed and normal economic activities resumed, human activities had changed into the new normal such as working and studying at home via an online platform, online shopping and ordering food as well as using online media for communication. The concentration of air pollutants during the study period is shown in Figure 2. The concentration of pollutants usually increased in the dry season, mainly resulting from the open burning of agricultural areas and forests [26]. Moreover, the meteorological factors in the dry season, such as high pressure, low wind speed, and little rain as well as thermal inversion, impacted the accumulation of pollutants in the area [27]. The concentration after the lockdown period in the five provinces is shown in Table 3. The average concentration of traffic-related pollutants ranged between 0.4 and 0.9 mg/m^3^ for CO and 12.2 and 16.7 µg/m^3^ for NO_2_. The concentrations were acceptable in the terms of the annual standard of Thailand at 57 µg/m^3^ for NO_2_ and the 8 h standard at 10.26 mg/m^3^ for CO. The overall concentration of traffic-related pollutants after the lockdown period showed an improvement of approximately 30% from that of the period before the lockdown. For particulate pollutants, the average concentration of PM_2.5_ ranged between 14.8 and 25.9 µg/m^3^. The concentration in KK was greater than the annual standard of PM_2.5_ at 25 µg/m^3^. The average concentration of PM_10_ ranged between 26.7 and 49.8 µg/m^3^. The concentrations were acceptable in terms of the annual standard of Thailand (50 µg/m^3^). The daily concentrations of PM_10_ and PM_2.5_ were usually higher than the 24 h standard (50 µg/m^3^ for PM_2.5_ and 120 µg/m^3^ for PM_10_) during winter and the transition from winter to the summer season because of the effects increased open burning sources and the meteorological impacts such as temperature inversion, high pressure and low wind speed [27]. The overall particulate pollutants of all provinces decreased by approximately 15% from the concentrations before the lockdown period. An exception was found in PK, where the particulate pollutants increased slightly. The average concentration of O_3_ ranged between 31.7 and 64.7 µg/m^3^, which is acceptable in terms of the 8 h standard of Thailand (140 µg/m^3^). The overall concentration of O_3_ slightly decreased by 4% from that before the lockdown period. In the case of SO_2_, the concentration before lockdown was similar to that after the lockdown. The average hourly concentration ranged between 1.8 and 7.9 µg/m^3^ with a maximum concentration of 13.6 µg/m^3^. This concentration was lower than that for 1 h at the Thailand standard for ambient SO_2_ at 780 µg/m^3^. The overall results for traffic-related pollutants showed that there was a significant long-term improvement in the new normal period in Thailand.

**Figure 3:**
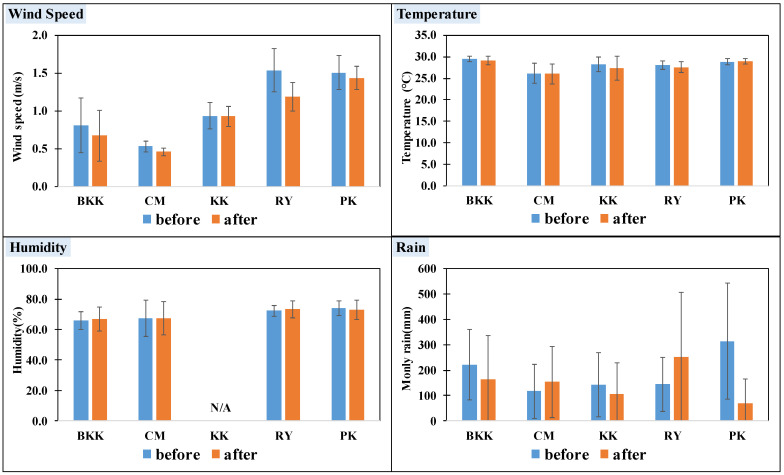
A comparison of meteorological factors before and after lockdown in Thailand.

Figure 3 presents a comparison of meteorological factors before and after lockdown in Thailand. The monthly average temperature, wind speed, humidity, and rain amount before lockdown were not significantly different (*p* < 0.05) from that after lockdown. The exception was observed for wind speed in BKK and RY which the wind speed after lockdown was 0.1–0.3 m/s lower than that before lockdown. Moreover, the monthly rain amount in PK decreased from before lockdown by 70%. In addition, the air pollutants data were collected over one year; therefore, the seasonal variation of meteorological parameters had a lower impact on the pollutant variation before and after lockdown.

### 3.2. Air Quality Index

**Figure 4:**
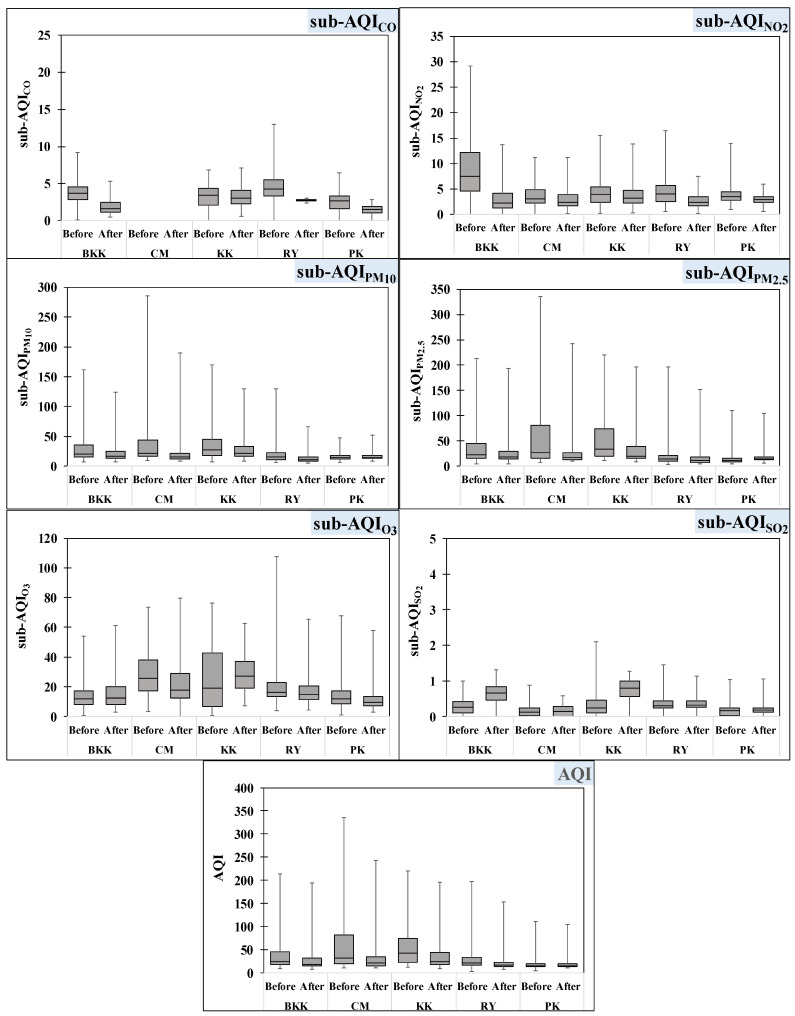
Boxplots of AQI and sub-AQI in Thailand.

The Thailand, air quality index (AQI) is usually used to report how air pollution impacts human health within a short time period. It is calculated from the criteria pollutants, including CO, NO_2_, SO_2_, O_3_, PM_10_ and PM_2.5_. Figure 4 shows boxplots of AQI and sub-AQI comparisons before and after the lockdown in each province. All provinces presented sub-AQI SO_2_ ranging between 0–2.1 which was very low compared to other pollutants. Therefore, a difference in sub-AQI between the periods before and after lockdown were scarcely observed for this pollutant. In metropolitan areas, AQI of BKK after lockdown ranged between 7.2 and 194 and the average AQI decreased by approximately 30% from that which occurred before the lockdown period. Sub-AQI after the lockdown period decreased by 55% for traffic-related pollutants (CO and NO_2_) and 26% for particulate matter (PM_10_ and PM_2.5_). However, the sub-AQI of O_3_ did not show any significant difference between both periods. For CM, the AQI after lockdown ranged between 9.9 and 243, which was an improvement of about 38% from that which occurred before the lockdown period. However, it was noted that CM usually had poorer air quality at the beginning of summer because of forest fires and burning of residue from agricultural areas [28]. Therefore, this study mainly compared the AQI changes in the rainy season and winter in order to evaluate the influence of the activities which changed in the new normal period. The overall sub-AQI after lockdown was reduced by about 20% from that which occurred before the lockdown period. Traffic-related pollutants showed significant improvement after the lockdown period. Even though the PM in the CM and BKK were generated from various sources (e.g., biomass burning, road dust, vehicle emissions, cooking, and the erosion of building products), traffic was a major source of pollution in the urban areas. An improvement of PM pollutants was also observed in the metropolitan province (BKK) [29,30,31].

The average AQI after lockdown of RY was 22.5 ± 19.6 which is an approximate decrease of 23% decreasing from the value before lockdown (29.3 ± 21.7). The sub-AQI of NO_2_ showed a significant decrease (50%) from the value before lockdown (0.56–16.5). Although CO was determined only in the rainy season after lockdown period, the sub-AQI value showed a 21% decrease compared to the same period before lockdown (0–13.0). Sub-AQI of O_3_, PM_10_ and PM_2.5_ after lockdown ranged from 4.3 to 65.2 for O_3_, 4.7 to 66.4 for PM_10_ and 4.3 to 152.4 for PM_2.5_. There was no significant difference in sub-AQI between, before, and after lockdown. Industrial activities (automotive production and burning of fossil fuels) and traffic densities were sources of PM in RY [32,33]. RY is the largest petroleum and petrochemical industrial complex in Thailand and it consists of petrochemical plants, oil refineries, coal-fired power plants, iron and steel plants, and plastic manufacturers. These factories were a source of volatile organic compounds (VOCs) which is the highest contributor to secondary ozone formation reactivity in RY [34,35]. Most factories in RY usually use machines for manufacturing, and there were few effects on production stemming from the new normal lifestyle. Therefore, the sub-AQI of industrial-emission pollutants after lockdown did not show any significant improvement from that which occurred before lockdown.

In KK, the AQI fell from 56.2 ± 42.5 before lockdown to 38.4 ± 34.1 after lockdown. Sub-AQI of CO and NO_2_ before lockdown were similar to the value after lockdown at 3.2 ± 1.1 for CO and 3.7 ± 2.3 for NO_2_. Sub-AQI values of PM_10_ and PM_2.5_ after lockdown decreased from that which occurred before lockdown by 21% and 38%, respectively. Sub-AQI O_3_ showed an increase of about 40% from that which occurred before lockdown. The Sub-AQI of PM contrasted with O_3_, which might have been due to PM reduction and strong solar intensity, which in turn led to greater photochemical activity and subsequent O_3_ production [36].

In the case of PK, the AQI after lockdown (17.5 ± 8.7) was similar to the value before lockdown (18.7 ± 9.9). Most air pollutants presented no significant differences in sub-AQI between before and after the lockdown periods. An exception was observed for sub-AQI CO (a traffic-related pollutant), for which the value decreased by 40% from that which occurred before the lockdown period (2.5 ± 1.1). The air quality in PK has normally been classified as excellent to satisfactory (AQI < 50) because the area is located on an island with high wind speeds and high rainfall which remove pollutants from the atmosphere. Therefore, the AQI in PK did not show any significant improvement when human activities changed to the new normal.

### 3.3. Health Risk Assessment of Exposure to Air Pollutants

The hazard quotient (HQ) was applied to estimate noncarcinogenic risks to humans from exposure to air pollutants by inhalation. The annual concentration of each study site was used for the calculation. Annual concentrations were then calculated for the HQ and HI values using Equations (1) and (4), with the assumption that the outdoor and indoor concentrations of air pollutants were equal because of the open house style. The HQ value of the study areas is presented in Table 4. The HQ values of traffic-related pollutants (CO and NO_2_) ranged between 0.2–0.7 for NO_2_ (less hazard) and 0.03–0.09 for CO (no hazard). The overall HQ values of traffic-related pollutants after lockdown improved by approximately 30% from the period before lockdown. The overall HQ values of PM (PM_10_ and PM_2.5_) ranged between 0.5 and 1.5, which indicated a less-to-moderate hazard level for the residents. The HQ values of PM in BKK and CM improved from a moderate hazard to a low hazard. In KK, the HQ value of PM after lockdown improved by 20% from that before lockdown, but there was a moderate hazard level for both periods. The HQ value of PM in RY and PK indicated a low hazard level for both the periods before and after lockdown. The overall HQ value of O_3_ (0.2–0.3) and SO_2_ (0.02–0.08) indicated a low hazard level and no hazard level, respectively.

**Figure 5:**
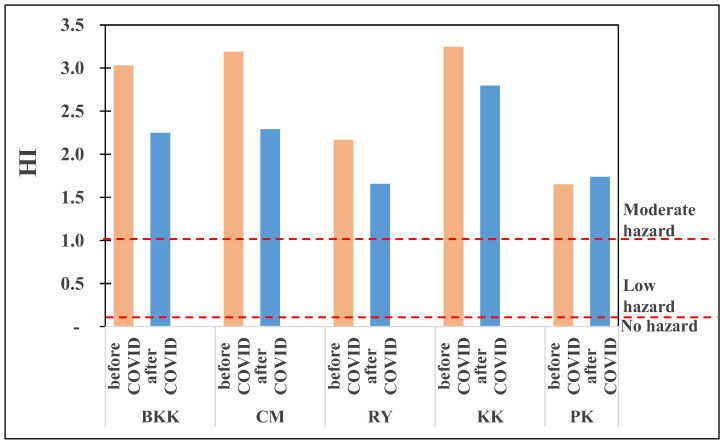
HI value in five provinces of Thailand.

In the case of the total health risks from exposure to the criteria pollutants, the HI value for all areas ranged from 1.7 to 3.3 (Figure 5), indicating moderate hazard levels for residents. The overall HI value after lockdown decreased from before lockdown by 23%. An exception was observed on PK, which did not demonstrate any significant improvement.

The HQ values of traffic-related pollutants after lockdown were only slightly reduced compared to before lockdown. Shen et al. [8] conducted a short-term study after the lockdown. Their results indicated that health risks from NO_2_ were 11.6% lower than recorded during COVID-lockdown measures in China, with no improvement in O_3_. In Malaysia, Othman and Latif [6] found that HI and HQ values of pollutant criteria, except O_3_ and PM_2.5_, decreased in the range between 3% and 81% as a result of short-term human activities which were controlled during the lockdown. Improvement of air pollutants during the COVID-19 lockdown reduced the morbidity attributed to air pollution, including respiratory diseases, cardiovascular disease, and chronic obstructive pulmonary disease (COPD) in tropical and subtropical countries by 5–35% [37]. However, results were evaluated shortly after lockdown measures and might not reflect long-term health effects.

The HI values in Thailand improved after the lockdown measures and were above the recommended limits for human health, while the HQ of PM was 68–75%, contributing to the HI value in Thailand. Other studies also found high-risk distribution of PM in metropolitan, industrial, and suburban areas of Thailand [38,39,40]. Results indicated a high potential of increased risk of respiratory diseases, COPD, lung cancer, and cardiovascular disease. Therefore, people should stay indoors or wear marks when outdoors to mitigate the risk of exposure to PM. Real-time air pollutant monitoring and rigorous measures to determine the sources of reduction in pollutants are required to improve health surveillance and air pollution control.

**Figure 6:**
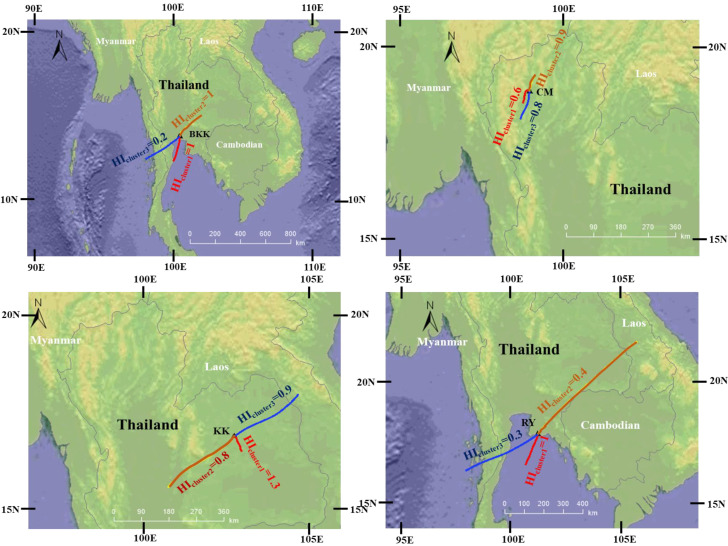

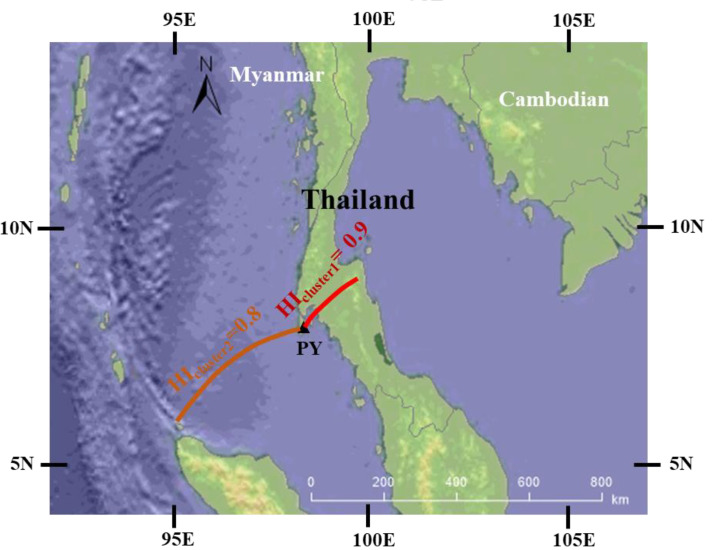
The clustered HI values in five provinces of Thailand.

In order to make a preliminary evaluation of the potential effects of pollutant sources on long-term health effects, a cluster analysis of air mass trajectories was applied for the assessment of health risks in the study areas. A total of 1096 backward trajectories were clustered, and the exposure frequency and the concentration of the pollutants in each cluster were then calculated to elucidate the human health risks (Table 5). Figure 6 presents the HI value of the clustered trajectories in each area. The HI value in BKK was 2.3 (a moderate hazard) and the values in clusters 1 and 2 were similar at 1 (a moderate hazard), while the value in cluster 3 was 0.2 (low hazard). The results indicated that the trajectories in clusters 1 and 2 significantly impacted human health risks in BKK. Even though the trajectories in cluster 1 were about twice those in cluster 2, the concentration of air pollutants in cluster 1 was lower than that in cluster 2, because cluster 1 originated from the sea (Gulf of Thailand) and passed through the industrial zone in the east of the province while cluster 2 passed through various sources such as traffic areas and open burning and industrial areas. Therefore, the HI value in cluster 1 was similar to that in cluster 2. For RY, the HI value was 1.7 (a moderate hazard). The HI value in cluster 1 was 1 (a moderate hazard) while the values in clusters 2 and 3 were 0.4 and 0.3, respectively. Although the concentration of air pollutants in cluster 2 (open burning sources) was the highest, the number of trajectories was only 18%. The pollutant concentration in cluster 1 was lower than that in cluster 2 but over 50% of the trajectories originated from the east coast of RY (cluster 1), which is an industrial zone. Therefore, the health risks of RY were mainly affected by cluster 1. In the case of KK, the HI value was 2.8 (a moderate hazard). The trajectories and pollutant concentration in cluster 1 were high compared to those in the other clusters. Cluster 1 presented the highest HI value (1.2; moderate hazard), while the HI values of clusters 2 and 3 were 0.7 and 0.9, respectively. This indicated that the health risk in KK was mainly influenced by the nearby area, which was mainly used for open burning. The results of BKK, RY and KK indicated that the clustered HI value clearly showed the path that possibly caused long-term health effects.

For PK, the HI value was 1.7 (a moderate hazard). The trajectories were divided into 2 clusters. Cluster 1 originated from the southern part of Thailand (community and forest fire sources), while cluster 2 originated from the Andaman Sea close to Sumatra Island, suggesting that a nearby community or neighboring country’s sources were possible sources of pollutants in cluster 2. The trajectories in cluster 2 (54%) were higher than those in cluster 1 (46%). The overall concentration of pollutants in cluster 1 was lower than those in cluster 2. Therefore, the HI value of cluster 1 (0.9; low hazard) was slightly greater than that in cluster 2 (0.8; less hazard). In CM, the concentration of pollutants in cluster 1 was the highest because it was generated by various sources (traffic, household and open burning) in the nearby areas including community, agricultural, and forest areas. Conversely, clusters 2 and 3 originated from nearby provinces and the air pollutants were also mainly generated from open burning sources. However, the trajectories in cluster 1 (23%) were the lowest; therefore, the HI value was not significantly different as it ranged between 0.6 and 0.9 (low hazard). The results for PK and CM indicated that all paths could possibly impact long-term human health effects.

The clustered HI values not only indicated the possible area and source of air pollutants, but also showed the non-cancer risk of the source, because this method used both trajectories and pollutant concentrations to calculate the HI value in each cluster. Using the clustered HI values was very helpful in establishing the primary source of a pollutant in order to determine air quality improvement.

## 4. Conclusions

This study evaluated long-term air quality after lockdown in Thailand, which resulted in a new normal of human behavior. Five provinces were selected to represent metropolitan areas (Bangkok and Chiang Mai), a suburban area (Khon Kaen), an industrial area (Rayong), and a tourism area (Phuket). The overall AQI value after lockdown decreased from before lockdown by 30%. However, a significant improvement in AQI value was not observed in Phuket, which had an excellent air quality level prior to lockdown, as well. Sub-AQI of air pollutants in the metropolitan areas presented a significant improvement of 30% from before lockdown. In a suburban area, there was no significant difference in sub-AQI of CO and NO_2_ between the periods before and after lockdown. In the case of an industrial area, a decrease in sub-AQI was observed in both CO and NO_2_ pollutants (34%) whereas those of other pollutants before lockdown were similar to those after lockdown because the production from factories was not impacted by the lockdown measures. These results were similar to those for the tourism area, in which sub-AQI of CO and NO_2_ was reduced by 30% due to a reduction in the number of visitors. The HI value before and after lockdown presented a moderate hazard level (1.7–3.3) from long-term exposure to air pollutants. The HQ value of PM was about 66–81%, which impacted the HI values in the study areas. The overall HI values after lockdown decreased by approximately 23% from those that occurred before lockdown because of the reduction in the concentration of air pollutants. The clustered HI values were useful to indicate the possible source areas of pollution. Likewise, the human health risks in each cluster of the trajectories were beneficial for determining the main sources of pollution in air quality so that improvements can be implemented in Thailand. However, it should be noted that those air pollutants in Thailand, particularly those emitted by transportation, have been seen to have improved as a result of the changes in human activities to the new normal lifestyle which were introduced in order to control the COVID-19 pandemic. Unfortunately, this is not a sustainable measure for air quality management. Additionally, other sources of pollution, namely, industrial and open burning, were also major sources of air pollutants in Thailand. Therefore, rigorous control measures for transportation and the control of other sources need to be further studied.

## Figures and Tables

**Table 1:** AQI value and air quality classifications.

Grade	AQI Value	Air Quality Level	Color
I	0–25	Excellent	
II	26–50	Satisfactory	
III	51–100	Moderate	
IV	101–200	Unhealthy	
V	>200	Very unhealthy	

**Table 3:** The concentration of air pollutants after the lockdown period in Thailand.

Pollutants		Concentration of Pollutants				
		BKK	CM	KK	RY	PK
CO	Average	0.4	N/A	0.7	0.6	0.4
(mg/m^3^)	Min–Max	0.1–1.0		0.1–1.5	0.5–0.6	0–0.7
NO_2_	Average	14.3	13.7	16.7	12.2	13.5
(µg/m^3^)	Min–Max	0.4–61.1	0.9–5.1	1.1–61.7	0.8–33.5	2.6–27.1
SO_2_	Average	6.6	1.8	7.9	3.9	2.1
(µg/m^3^)	Min–Max	0–13.6	0–6.0	0–13.4	0–11.8	0–11.0
O_3_	Average	38.2	50.2	64.7	44.5	31.8
(µg/m^3^)	Min–Max	8.6–100	1.2–125.2	20.6–118	11.6–110.2	7.6–103.7
PM_10_	Average	39.8	40.1	49.8	26.7	32.7
(µg/m^3^)	Min–Max	12.8–152.8	16.2–168.2	19.3–137.8	8.6–90.8	18.4–92.0
PM_2.5_	Average	22.4	24.5	25.9	14.8	16.5
(µg/m^3^)	Min–Max	5.1–100.8	8.8–131.2	9.0–87.5	3.4–67.7	5.7–61.3

**Table 4:** HQ value of air pollutants in Thailand.

Provinces		HQ Value of Pollutants					
		HQ_CO_	HQ_NO_2__	HQ_PM_2.5__	HQ_PM_10__	HQ_O_3__	HQ_SO_2__
BKK	before COVID	0.07	0.7	1.1	1.0	0.2	0.03
	after COVID	0.04	0.3	0.9	0.8	0.2	0.07
CM	before COVID	N/A	0.3	1.5	1.2	0.3	0.02
	after COVID	N/A	0.2	0.9	0.8	0.3	0.02
RY	before COVID	0.09	0.4	0.7	0.7	0.3	0.04
	after COVID	0.05	0.2	0.6	0.5	0.2	0.04
KK	before COVID	0.06	0.3	1.4	1.2	0.3	0.04
	after COVID	0.06	0.3	1.0	1.0	0.3	0.08
PK	before COVID	0.05	0.3	0.5	0.6	0.2	0.02
	after COVID	0.03	0.2	0.6	0.6	0.2	0.02

**Table 5:** Concentration of pollutants and frequency exposure in each cluster for HQ calculation.

Provinces	Concentration/ Exposure Frequency (EF)	Concentration			
		Cluster 1	Cluster 2	Cluster 3	Overall
BKK	CO (mg/m^3^)	0.3	0.7	0.3	0.4
	NO_2_ (µg/m^3^)	12.6	28.8	6.5	14.2
	PM_2.5_ (µg/m^3^)	20.2	30.7	13.8	22.4
	PM_10_ (µg/m^3^)	35.3	54.0	27.0	39.8
	O_3_ (µg/m^3^)	36.0	47.8	23.9	38.1
	SO_2_ (µg/m^3^)	7.1	3.7	8.4	6.5
	Number of trajectories	567	344	185	1096
	EF (d/y)	189	115	62	365
CM	CO (mg/m^3^)	N/A	N/A	N/A	N/A
	NO_2_ (µg/m^3^)	14.8	14.0	12.6	13.7
	PM_2.5_ (µg/m^3^)	27.6	24.1	23.2	24.5
	PM_10_ (µg/m^3^)	44.4	39.0	38.7	40.1
	O_3_ (µg/m^3^)	54.1	48.0	49.6	50.1
	SO_2_ (µg/m^3^)	1.7	2.0	1.5	1.7
	Number of trajectories	257	383	456	1096
	EF (d/y)	86	128	152	365
KK	CO (mg/m^3^)	0.7	0.4	0.8	0.7
	NO_2_ (µg/m^3^)	18.5	12.3	20.7	16.8
	PM_2.5_ (µg/m^3^)	28.2	20.8	28.4	25.9
	PM_10_ (µg/m^3^)	52.1	41.6	55.6	49.8
	O_3_ (µg/m^3^)	60.7	65.9	68.7	64.8
	SO_2_ (µg/m^3^)	0.7	0.4	0.8	0.7
	Number of trajectories	446	340	310	1096
	EF (d/y)	149	113	103	365
RY	CO (mg/m^3^)	0.5	N/A	0.6	0.5
	NO_2_ (µg/m^3^)	13.1	16.2	8.1	12.3
	PM_2.5_ (µg/m^3^)	16.4	19.6	8.2	14.8
	PM_10_ (µg/m^3^)	28.4	34.1	18.1	26.7
	O_3_ (µg/m^3^)	45.2	53.2	37.1	44.5
	SO_2_ (µg/m^3^)	3.7	3.6	4.3	3.9
	Number of trajectories	605	199	292	1096
	EF (d/y)	201	66	97	365
PK	CO (mg/m^3^)	0.3	0.3		0.3
	NO_2_ (µg/m^3^)	13.9	13.1		13.5
	PM_2.5_ (µg/m^3^)	19.3	14.0		16.5
	PM_10_ (µg/m^3^)	37.5	28.6		32.7
	O_3_ (µg/m^3^)	42.6	22.7		31.8
	SO_2_ (µg/m^3^)	2.1	2.3		2.2
	Number of trajectories	504	592		1096
	EF (d/y)	168	197		365

## Data Availability

The Global Data Assimilation System (GDAS) and meteorological data (1° × 1°) during the study were obtained from ftp://arlftp.arlhq.noaa.gov/pub/archives/gdas1/ (accessed on 25 June 2022). TrajStat (trajectory Statistics) which was developed by Yaqiang Wang in 2008 was used for calculation in this study. It was available at: http://meteothink.org/downloads/index.html (accessed on 20 June 2022).
